# m6A Methylation in Cardiovascular Diseases: From Mechanisms to Therapeutic Potential

**DOI:** 10.3389/fgene.2022.908976

**Published:** 2022-06-28

**Authors:** Longbo Li, Nannan Xu, Jia Liu, Zhenzhen Chen, Xu Liu, Junnan Wang

**Affiliations:** Department of Cardiology, Second Hospital of Jilin University, Changchun, China

**Keywords:** epigenetics, cardiovascular pathophysiology, cardiovascular diseases, m6A demethylase, m6A methyltransferase, m6A

## Abstract

Cardiovascular disease (CVD) is a leading cause of morbidity and mortality worldwide. Recent studies have shown that n6-methyladenosine (m6A) plays a major role in cardiovascular homeostasis and pathophysiology. These studies have confirmed that m6A methylation affects the pathophysiology of cardiovascular diseases by regulating cellular processes such as differentiation, proliferation, inflammation, autophagy, and apoptosis. Moreover, plenty of research has confirmed that m6A modification can delay the progression of CVD via the post-transcriptional regulation of RNA. However, there are few available summaries of m6A modification regarding CVD. In this review, we highlight advances in CVD-specific research concerning m6A modification, summarize the mechanisms underlying the involvement of m6A modification during the development of CVD, and discuss the potential of m6A modification as a therapeutic target of CVD.

## 1 Introduction

Cardiovascular disease (CVD), including cardiac diseases and vascular diseases, is a leading cause of morbidity and mortality worldwide and regarded as a significant public health problem (Leong et al., 2017; Andersson and Vasan, 2018). Over the past decade, there is a tremendous development in the diagnosis and treatment of CVD, however, it is still challenging to approach the rising morbidity and mortality rates in CVD patients. A big problem is that the mechanisms regulating CVD onset and progression are still not entirely clear. It has been realized that genetics and environments play great role in the onset and progression of CVD, and with the help of molecular biology technique, we could explore the role of genetics in the pathophysiology of CVD in the cellular and molecular level and develop genetic therapy agents for CVD (Ylä-Herttuala and Baker, 2017). However, neither the genetics nor the environments are sufficient to fully explain the onset and progression of CVD. Recent discoveries revealed that environments could alter gene expression without genetic changes, indicating that there is a link between genetics and environments (Prasher et al., 2020). For example, hypoxia could change the expression of hypoxia-related genes without changing DNA sequence of these genes; this regulation of gene expression without the change of DNA sequences in response to environmental factors is called epigenetics, through which we can explore the link between genetics and environments (Jones et al., 2016).

Epigenetics, including histone modification, DNA methylation, RNA methylation and non-coding RNA molecules, has attracted tremendous research interests due to its special regulation of gene expression. Latest research revealed that epigenetic modifications is heritable, reversible, and regulated by a serious of proteins, such as writers (that deposit them), readers (to interpret them) and erasers (to remove them) (Cavalli and Heard, 2019). Additionally, more and more researches confirmed that epigenetics plays a great role in the onset and development of CVD through regulating the expression of CVD-related genes, which further affect cell function and contribute to the progression of CVD (Prasher et al., 2020). The role of histone modification, DNA methylation and non-coding RNA in the onset and development of CVD have been well studied, however, m6A modification, the most common and abundant epigenetic modification in eukaryotic RNA (Chen Y.-S. et al., 2021; Shen Z.-J. et al., 2021), has attracted tremendous research interests just in recent years. Emerging studies confirmed that m6A modification play significant role in the pathophysiology of CVD, such as the proliferation, autophagy, apoptosis, and inflammation of cardiovascular system cells, through post-transcriptional regulation of CVD-related RNA. Regulation of m6A methylation has been proven to reverse the progression of CVDs and may be a therapeutic target for the treatment of CVDs (Zhao K. et al., 2020). However, given this large number of researches, few reviews have been published regarding the relationship between m6A modification and CVD, despite the ability of the latter to cause major acute cardiovascular events. To achieve an in-depth understanding of the function and regulatory mechanism of m6A modification regarding CVD, it is necessary to summarize existing knowledge of m6A modification in relation to CVD. Such a summary will contribute to the prevention, diagnosis, and treatment of CVD.

To this end, herein, we review the role of m6A modification in CVD. First, we introduce the molecular mechanism, regulation, and biological function of m6A modification. Second, we summarize the mechanisms of m6A modification that are involved in the cardiovascular pathophysiology. Third, we discuss the potential role of m6A modification in CVD.

## 2 m6A Methylation

### 2.1 Molecular Mechanism of m6A Methylation

Methylation of adenine at the 6N position, commonly called m6A methylation (Wu et al., 2021), was first discovered in 1974 in the messenger RNA (mRNA) of mammalian cells (Desrosiers et al., 1974). Since its discovery, only few functional studies have been conducted on m6A modification because of a lack of reliable detection technologies. In 2012, however, a breakthrough came in the form of m6A sequencing, which can identify target transcriptions that have undergone m6A modification (Dominissini et al., 2012; Meyer et al., 2012).

m6A modification is characterized by its dynamic reversibility, wide distribution, and highly conservative nature. As the most abundant RNA modification in eukaryotes, m6A modification is widely found in almost all types of RNA (Wu S. et al., 2020), such as mRNA, transfer RNA (tRNA), circular RNA (circRNA), long non-coding RNA (lncRNA) RNA, and ribosomal RNA (rRNA), and more than 25% of human transcripts are modified by m6A (Wu et al., 2021). m6A modification is not static but is instead dynamically regulated by m6A methyltransferases and demethylases (Liu et al., 2014; Zhang W. et al., 2021). Additionally, this type of epigenetic modification is enriched near stop codons and in 3’ untranslated regions (UTRs). Moreover, it is distributed across the highly conserved RRACH sequence (R = A or G and H = A, U, or C) (Xia et al., 2021), where adenosine is modified to m6A.

### 2.2 Regulation of m6A Methylation Modification

**FIGURE 1:**
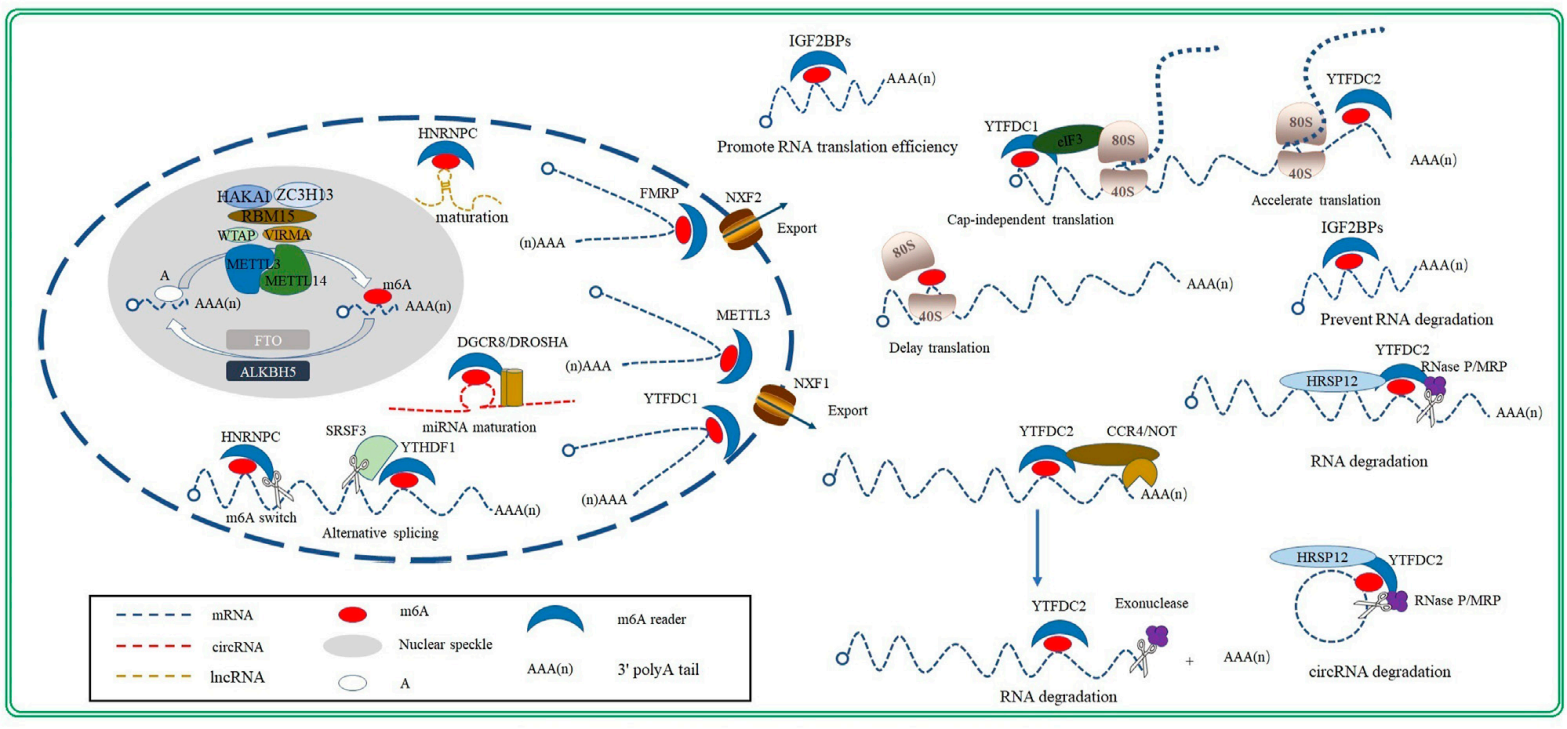
Regulation and biological function of m6A modification.

m6A methylation is a dynamic and reversible process that is primarily regulated by three proteases: m6A methyltransferase (writer), m6A demethylase (eraser), and m6A RNA-binding proteins (reader; Figure 1). The m6A methyltransferase complex catalyzes m6A modification, m6A demethylase removes the methyl group and modifies m6A to A, and m6A RNA-binding proteins recognize and combine m6A-methylation sites to regulate RNA mentalism. The combined action of m6A methyltransferase and m6A demethylase ensures that m6A RNA methylation remains balanced in cells. Therefore, many functional studies on m6A modification have been performed by either knocking down or overexpressing m6A methyltransferase or demethylase (Shen et al., 2015; Dorn et al., 2019; Ma et al., 2020; Wang et al., 2020; Wang P. et al., 2021; Takemoto et al., 2021; Xia et al., 2021).

#### 2.2.1 m6A Methyltransferase (Writer)

The m6A methyltransferase complex, consisting of methyltransferase-like3 (METTL3), methyltransferase-like14 (METTL14), and Wilms tumor 1associated protein (WTAP), specifically recognizes the RRACH sequence in RNA and catalyzes the modification of adenosine to m6A (Liu et al., 2014; Figure 1). METTL3 is a key enzyme within this complex; it possesses an S-adenosylmethionine (SAM)-binding domain that can transfer methyl groups from the m6A-methylation substrate to the sixth N of adenosine. Moreover, METTL3 is the only catalytic subunit in the m6A methyltransferase complex; therefore, its deletion inactivates the complex (Geula et al., 2015). The sequence homology between METTL14 and METTL3 can reach as high as 43%. However, unlike METTL3, METTL14 has no catalytic activity. METTL14 has an arginine-glycine-glycine domain at its C-terminus, which provides a platform for an RNA and greatly improves the complex’s stability and catalytic ability (Liu et al., 2014; Schöller et al., 2018; Chen Y.-S. et al., 2021). Moreover, METTL3 and METTL14 form the core of the m6A methyltransferase complex (Liu et al., 2014) and localizes these complex to the nuclear speckle, which is enriched in splicing factors (Liu et al., 2014; Alarcón et al., 2015a; Liu et al., 2015; Wang et al., 2016).

Similar to METTL14, WTAP does not exhibit methyltransferase activity; however, its knockdown significantly reduces the level of m6A modification in RNA, to an even more severe degree than METTL14 knockdown. This suggests that WTAP is essential to m6A modification (Duan et al., 2019). Furthermore, studies have confirmed that WTAP helps localize the m6A methyltransferase complex to the nuclear speckle and recruits it to the substrate RNA (Ping et al., 2014; Zhang W. et al., 2021; Wu et al., 2021).

Studies have also identified other protein components, such as Vir-like m6A methyltransferase-associated protein (VIRMA), RNA binding motif protein15 (RBM15), Cbl photo oncogene like1 (HAKAI), and zinc finger CCCH-type containing 13 (ZC3H13), in the m6A methyltransferase complex by co-immunoprecipitation (Liu et al., 2014; Yue et al., 2018; Zhu et al., 2020; Wang P. et al., 2021, Wang et al., 2021 J.; Zhang et al., 2021b; Figure 1). Moreover, studies have discovered that the ablation of VIRMA, RBM15, HAKAI or ZC3H13decreased the degree of m6A methylation in RNAs, indicating that these proteins are subunits of the m6A methyltransferase complex. However, their roles in the catalytic process remain to be studied (Patil et al., 2016; Růžička et al., 2017; Wen et al., 2018).

#### 2.2.2 m6A Demethylase (Eraser)

Two m6A demethylases have been identified in eukaryotes: FAT mass and obesity-associated protein (FTO) and ALKB homologue5 protein (ALKBH5) (Zheng et al., 2013). They are both Fe(II)/α-ketoglutarate-dependent dioxygenases, and both are members of the ALKBH family (Zaccara et al., 2019). Additionally, FTO and ALKBH5 are both localized to the nucleus, and their knockdown significantly increases m6A-methylation levels (Shen et al., 2015; Shen W. et al., 2021; Zhao Y. et al., 2021). The discovery of m6A demethylases revealed the reversibility of m6A modification, indicating that m6A modification is a dynamically regulated process (Wu et al., 2021).

*FTO* (also referred to as ALKBH9) was first identified as an obesity-related gene; it exhibits dioxygenase activity that oxidizes m6A to form an N6-hydroxymethyladenosine intermediate, which is further oxidized to N6-formyladenosine. Both N6-hydroxymethyladenosine and N6-formyladenosine remove formaldehyde or formic acid molecules and form adenylate to complete RNA demethylation (Fu et al., 2013). Unlike FTO, ALKBH5 directly demethylates the m6A site; it skips the production of intermediates (Zheng et al., 2013; Figure 1). FTO and ALKBH5 both are essential to cardiac homeostasis. Studies have confirmed that FTO and ALKBH5 play a significant role in the development of embryo heart and cardiovascular disease, such as atherosclerosis, coronary heart disease (CHD) and heart failure (Mathiyalagan et al., 2019; Song et al., 2019; Shen W. et al., 2021).

#### 2.2.3 m6A RNA-Binding Proteins (Reader)

The m6A RNA-binding proteins, called readers, recognize and bind to the m6A methylation sites to regulate RNA mentalism (Wu et al., 2021). The most well-known m6A RNA-binding protein is a member of the large YTH family, which comprises the YTH domain-containing family protein 1–3 (YTHDF1/2/3) and YTH domain-containing protein 1–2 (YTHDC1/2) subfamilies. Members of the YTH family possess a YTH domain, which constitutes an aromatic pocket structure that can recognize and bind to m6A-methylation sites. Within the YTHDF subfamily, YTHDF1 can increase the ribosome-occupancy rate and improve translational efficiency by directly interacting with translation-initiation factors, while YTHDF2 facilitate the degradation of mRNA modified by m6A (Sheth and Parker, 2003). Studies have demonstrated that YTHDF2 knockout increases the stability of target mRNAs, which can prolong their lifespan by ∼30% (Wang X. et al., 2014; Schwartz et al., 2014; Wang et al., 2015). Furthermore, YTHDF3 acts as a “buffer” for YTHDF1 and YTHDF2, as it binds to m6A-methylated RNA that has entered the cytoplasm. It can promote the combination of YTHDF1/YTHDF2 and target mRNA, thus accelerating the translation or degradation of the target transcript induced by YTHDF1 or YTHDF2 (Shi et al., 2017).

YTHDC1, as a member of the YTHDC subfamily, recognizes m6A-methylation sites and enhances the binding of target transcripts to serine- and arginine-rich splicing factor 3 (SRSF3) and nuclear RNA-export factor 1 (NXF1). In this way, it promotes the nuclear export of mRNA in an m6A-dependent manner (Roundtree et al., 2017). Additionally, YTHDC2 regulates RNA translation and decay by recognizing m6A-methylation sites on target RNA (Mao et al., 2019).

Insulin-like growth factor 2 mRNA-binding proteins (IGF2BPs), including IGF2BP1, IGF2BP2, and IGF2BP3, is another important family of m6A RNA-binding proteins. Functionally, IGF2BPs promote the stability and the translation efficiency of target m6A-modified mRNA (Zhao Y. et al., 2020). Advances in research have uncovered new m6A RNA-binding proteins, such as heterogeneous nuclear ribonucleoprotein C (HNRNPC) and heterogeneous nuclear ribonucleoprotein A2/B1 (HNRNPA2B1) (Wu et al., 2021). Interestingly, the m6A methyltransferase METTL3 also functions as an m6A RNA-binding protein under certain circumstances (Alarcón et al., 2015a; Liu et al., 2015; Zhou et al., 2019).

### 2.3 m6A Methylation and Post-Transcriptional Regulation

m6A modification, as well as other types of epigenetic, regulate gene expression by affecting the physiological properties of RNA transcripts, including their charge, base pairing, secondary structure, and protein–RNA interactions (Yang et al., 2018). Furthermore, m6A modification participates in almost every stage of the RNA life cycle, and it determines the fate of m6A-methylated RNA (Kmietczyk et al., 2019; Zhao K. et al., 2020).

#### 2.3.1 Post-Transcriptional Regulation of mRNAs by m6A Methylation

##### 2.3.1.1 mRNA Maturation

The level of m6A modification is proportional to the amount of mature mRNA in the cytoplasm. In fact, the down-regulation of m6A modification has been shown to significantly decrease the amount of mature RNA in the cytoplasm, suggesting that m6A modification participates in the process of mRNA maturation. Splicing is a key process during mRNA maturation in eukaryotic cells. Both m6A methyltransferases and demethylases are located in nuclear speckles, where splicing occurs. This suggests that m6A modification and mRNA splicing may be connected in some way. In fact, m6A modification was initially recognized as a splicing regulator (Zhao et al., 2017); and its interaction with splicing factors may represent one of the mechanisms through which m6A modification influences mRNA splicing. Studies have confirmed that m6A modification in alternatively spliced exons promotes alternatively spliced retention by recruiting SRSF3 and blocking the binding of the exon-skipping factor SRSF10, after being recognized by YTHDC1 (Roignant and Soller, 2017; Figure 1). The down-regulation of m6A modification by the knockout of m6A methyltransferases has been shown to reduce the splicing efficiency rate (Wei et al., 1975; Csepany et al., 1990; Gerken et al., 2007). Interaction with RNA-binding proteins is another mechanism of m6A modification during the regulation of mRNA splicing. HNRNPC is an RNA-binding protein that promotes mRNA processing and maturation by binding to a single-stranded RNA-binding motif hidden in pre-mRNA molecules. m6A modification make this single-stranded RNA-binding motif exposed to HNRNPC by weakening the base pairing. This allows HNRNPC to bind to target mRNA while promoting pre-mRNA processing and maturation (Figure 1); this process is called “m6A switch” (Liu et al., 2015).

Mature mRNAs have a 5′ cap and a 3′ tail to initiate translation and avoid nuclease-mediated degradation. Methylphosphate capping enzyme (MEPCE) is a crucial enzyme required for 5′ capping. Warda et al. (2017) performed co-immunoprecipitation and discovered that MEPCE is a part of the m6A methyltransferase complex. This implies that m6A methylation may be involved in 5′ capping of mRNAs (Warda et al., 2017). Furthermore, more than 70% of m6A methylations are located in the last exons of mRNAs, which affects the position of the 3′ polyA tail. This is evidenced by the knockout of m6A methyltransferases or m6A RNA-binding proteins, altering the position of the 3′ polyA mRNA tail and modifying the 3′-UTR (Beal et al., 2007; Warda et al., 2017).

##### 2.3.1.2 Nuclear Export

Mature mRNA must translocate from the nucleus to the cytoplasm to perform its myriad functions; however, the knockout of m6A methyltransferases can hinder the nuclear export of mRNA (Fustin et al., 2013; Lesbirel et al., 2018). It has been confirmed that m6A modification facilitate the nuclear export of mRNA through the nuclear export complex, or via export complex factors. Specifically, m6A modification recruits the transcription–export (TREX) complex through METTL3; TREX then recruits NXF1, thus promoting the nuclear export of mRNA (Zheng et al., 2013; Lesbirel et al., 2018; Figure 1). Similarly, the reader proteins YTHDC1 (Roundtree et al., 2017) and FMRP (Lai et al., 2006; Hsu et al., 2019) promote mRNA export by transporting m6A-methylated mRNAs to the nuclear export receptors NXF1 and NXF2 (Figure 1), respectively.

##### 2.3.1.3 RNA Translation

Eukaryotic initiation factor (eIF) 4E, which is a 7-methylguanosine-containing mRNA cap-binding protein, can recruit eIF3 and initiate cap-dependent translation. However, m6A located in the 5′ UTR of mRNA can be combined by ribosomes as a substitute for the 5′ cap. In this way, it can promote the initiation of cap-independent translation. Specifically, in the absence of eIF4E, the 5’ UTR of m6A binds to eIF3 through YTHDF1 (Wang et al., 2015; Lin et al., 2016), thus enabling mRNA to bind to ribosomes. This allows the consequent initiation of cap-independent translation (Berulava et al., 2020; Figure 1).

m6A modification exert a dual effect on the translation elongation of mRNA. On the one hand, m6A modification slows the binding of the ternary complex to the ribosomal A site and then delay translation elongation (Choi et al., 2016; Figure 1). On the other hand, the m6A RNA-binding protein YTHDC2 functions as an RNA helicase; it recognizes an m6A-methylation site and then accelerates translation elongation by unwinding the secondary structure of mRNA (Liu and Zhou, 2021; Figure 1). T Therefore, a balanced m6A-methylation process forms the basis of translation elongation, whereas an imbalance can influence the translation process.

Approximately >25% of m6A modifications in the entire transcriptome occur in the 3′ UTR, near the mRNA stop codon (Meyer et al., 2012), suggesting that m6A modifications may also participate in the termination of mRNA translation. However, the exact mechanism of the m6A modification’s role in translation termination requires further study.

##### 2.3.1.4 mRNA Degradation

The function that m6A modification plays in the regulation of mRNA degradation was identified in 1978. The half-lives of m6A methylated mRNA were found to be significantly shorter than those of mRNA without m6A modification. Following studies confirmed that the up-regulation of m6A modification could facilitate mRNA degradation. Moreover, further studies have since revealed that YTHDF2 induced m6A-mediated mRNA degradation. YTHDF2 binds to the m6A-modification sites of mRNA with its C-terminus, whereas its N-terminus localizes mRNA to P body where RNA is degraded (Sheth and Parker, 2003). Currently, there are two known pathways for m6A-methylation-mediated mRNA degradation: the YTHDF2–carbon catabolite-repression 4 (CCR4)/negative on TATA-less (NOT) complex pathway (Figure 1) and the YTHDF2–heat-responsive protein 12 (HRSP12)–ribonuclease (RNase) P/mitochondrial RNA-processing (MRP) endonuclease complex pathway (Figure 1). YTHDF2 facilitates the deadenylation of mRNA by recruiting the CCR4/NOT complex, thereby promoting rapid, exonuclease-mediated mRNA degradation (Wang X. et al., 2014). On the other hand, m6A-methylated target mRNA containing HRSP12-binding sites have been found to be preferentially bound by HRSP12. This consequently promotes the binding of YTHDF2 to target mRNA. Thereafter, YTHDF2 accelerates mRNA degradation by recruiting the RNase P/MRP complex, which mediates mRNA degradation via its endonuclease activity (Park et al., 2019; Figure 1).

#### 2.3.2 Non-Coding RNA

In addition to directly acting upon mRNA, m6A modification also regulates gene expression by acting on non-coding RNA. The DiGeorge syndrome critical region 8 (DGCR8)/Drosha ribonuclease III (DROSHA) complex acts as a crucial cleavage enzyme that participates in the processing and maturation of microRNA (miRNA). Specifically, m6A modification promotes the binding of primary-miRNA (pri-miRNA) with DGCR8, which further recruits DROSHA to promote maturation of pri-miRNA (Alarcón et al., 2015b; Figure 1). Similarly, m6A modification in the hairpin region of lncRNA also facilitates their processing and maturation by loosening the hairpin structure and facilitating their binding to HNRNPC via the m6A switch mechanism mentioned above (Figure 1). The closed-loop structure of circular RNA (circRNA) offers protection from exonuclease degradation; nevertheless, m6A can accelerate its degradation through the YTHDF2–HRSP12–RNase P/MRP pathway (Figure 1). Notably, m6A modification also affects the function of non-coding RNA. For example, miRNA can direct the silencing complex to degrade target mRNA. However, IGF2BPs can block target mRNA from binding to miRNA by recruiting human antigen R, thereby improving mRNA stability (Fan and Steitz, 1998; Peng et al., 1998).

As mentioned above, m6A modification participates in almost every stage of RNA metabolism, including RNA maturation, nuclear export, translation, and degradation ^48^. This indicates that m6A modification has the potential to prevent and treat diseases by regulating the fate and function of RNA.

## 3 m6A Methylation and Cardiovascular Pathophysiology

The process of m6A methylation is critical for the development, differentiation, homeostatic maintenance, and stress response of the cardiovascular system. Many m6A modifications are present in the heart (Dorn et al., 2019), comprising approximately a quarter of the total transcripts in healthy mouse and human hearts (Berulava et al., 2020). A high-throughput sequencing study discovered that m6A-methylated RNAs are widely involved in myocardial development, energy metabolism, stress response, and myocardial remodeling in human and mouse hearts (Berulava et al., 2020). Normal m6A modification levels maintain cardiac homeostasis, but METTL3 overexpression can disturb cardiac homeostasis and induce cardiomyocyte hypertrophy (Dorn et al., 2019). Therefore, regulating m6A methylation may be a novel approach for reversal of CVD progression.

### 3.1 Regulation of Cell Proliferation

Studies have confirmed that m6A methylation promotes tumorigenesis and tumor progression by regulating cell proliferation through the AKT, p38/ERK, and Wnt/β-catenin pathways (Liu et al., 2018; Deng et al., 2019; Li Y. et al., 2021). And latest research suggest that m6A methylation also plays great roles in cellular proliferation of cardiovascular system (Liu et al., 2018; Deng et al., 2019; Li Y. et al., 2021). One study found that ALKBH5 knockdown inhibits cardiomyocyte proliferation in neonatal mice, whereas overexpression promotes cardiomyocyte proliferation following myocardial infarction. Furthermore, ALKBH5 promotes YAP translation by enhancing the stability of YTHDF1 mRNA in an m6A dependent manner (Han et al., 2021b), which enables cardiomyocytes to re-enter the cell cycle and proliferate (Table 1). Upregulation of WTAP expression in vascular smooth muscle cells (VSMCs) promotes p16 expression by increasing m6A methylation level of *p16* mRNA and inhibits cell proliferation of VSMCs (Zhu et al., 2020; Table 1). Similarly, FTO knockdown increases m6A methylation of SM22α; then the m6A RNA-binding protein IGF2BPs recognizes m6A-methylated SM22α mRNA and enhances its stability and expression, thus inhibiting the proliferation and migration of VSMCs in type 2 diabetes patients, while also improving intimal hyperplasia (Zhang et al., 2022; Table 1). Of note, cell proliferation plays different roles in various CVDs. For example, cell proliferation reduces myocardial infarct size and improves cardiac function; however, hyperproliferation of VSMCs can cause atherosclerosis. Therefore, it may be inefficient to regulate cell proliferation by altering m6A methylase or demethylase expression. Alternatively, precise regulation of m6A methylation levels using gene editing technologies may be a good approach to the regulation of cell proliferation of cardiovascular system (Liu X.-M. et al., 2019; Rau et al., 2019).

**TABLE 1:** The role of m6A methylation in cardiovascular pathophysiology.

Cardiovascular Pathophysiology	m6A-Related molecules	Function	Target Gene	Mechanism	References
Regulation of cell proliferation	WTAP	Writer	p16	Upregulation of WTAP expression in VSMCs promotes p16 expression by increasing m6A methylation level of p16 mRNA and inhibits cell proliferation of VSMCs	Zhu et al. (2020)
	ALKBH5	Eraser	YTHDF1	ALKBH5 promotes YAP translation by enhancing the stability of YTHDF1 mRNA in an m6A dependent manner, which enables cardiomyocytes to re-enter the cell cycle and proliferate	Han et al. (2021b)
	FTO	Eraser	SM22α	FTO knockdown inhibits the proliferation and migration of VSMCs in type 2 diabetes patients by SM22α	Zhang et al. (2022)
	IGF2BP2	Reader	SM22α	IGF2BPs inhibits the proliferation and migration of VSMCs in type 2 diabetes patients by enhancing SM22α mRNA stability and expression as m6A RNA-binding protein	Zhang et al. (2022)
Regulation of cell differentiation	METTL3	Writer	—	METTL3 promote the differentiation of mesodermal cells into cardiomyocytes	Batista et al. (2014)
	ALKBH5	Eraser	—	Upregulating the level of RNA m6A methylation by inhibiting the expression of ALKBH5 can cause cardiomyocyte differentiation disorder	Han et al. (2021c)
	YTHDF1	Reader	Cardiomyocyte-specific gene	Knockout of YTHDF1 impairs cardiomyocyte differentiation and downregulates cardiomyocyte-specific gene expression	Wang et al. (2021c)
	YTHDF3	Reader	Cardiomyocyte-specific genes	Knockout of YTHDF3 accelerates the differentiation of stem cells into cardiomyocytes by promoting the expression of cardiomyocyte-specific genes	Wang et al. (2021c)
Regulation of cell autophagy	METTL3	Writer	TFEB	METTL3 impairs autophagic flux by accelerating the degradation of the TFEB mRNA and inhibiting its expression	Song et al. (2019)
			mic-20b	METTL3 inhibits H/R-induced autophagy by promoting maturation of pri-miR-20b	Lu et al. (2020)
	FTO	Eraser	ULK1	FTO promotes autophagy by prolonging the half-life of the ULK1 mRNA in an m6A dependent manner	Jin et al. (2018)
Regulation of cell apoptosis	METTL3	Writer	miR-25-3P	METTL3 activates the P13K/Akt pathway in cardiomyocytes and reduces H/R-induced apoptosis in cardiomyocytes by promoting the maturation of miR-25-3P and miR-873-5p	Zhao et al. (2021a)
			miR-873-5p		
	FTO	Eraser	Mhrt	FTO inhibits the H/R-induced apoptosis of cardiomyocytes by promoting the expression of Mhrt	Shen et al. (2021a)
Regulation of inflammatory cells	ALKBH5	Eraser	IL6	ALKBH5 downregulate inflammatory response by inhibiting the nuclear export of IL6 mRNA	Zhao et al. (2020a)
	METTL3	Writer	STAT1	METTL3 promotes an inflammatory response induced by ox-LDL by upregulating the m6A methylation of STAT1	Li et al. (2022)
	FTO	Eraser	—	FTO silencing promotes an inflammatory response by inducing the transformation of macrophages into M1-type proinflammatory macrophages	Hu et al. (2019)

### 3.2 Regulation of Cell Differentiation

Presently, studies have confirmed that m6A methylation plays several roles in post-transcriptional modification during stem cell differentiation, and it determines the fate of embryonic stem cells (Batista et al., 2014; Wang Y. et al., 2014; Slobodin et al., 2017; Wen et al., 2018; Kwon et al., 2019; Han et al., 2021c; Wang S. et al., 2021). In fact, complete knockout of the m6A methyltransferases METTL3 (Geula et al., 2015) and METTL14 (Yoon et al., 2017) leads to embryonic death, suggesting that m6A methylation is critical for embryonic development and organ differentiation. During embryonic development, the heart develops from mesodermal cells. In this regard, a study has found that when mesodermal cells differentiate into cardiomyocytes, the level of RNA m6A methylation is significantly upregulated. Furthermore, an *in vitro* differentiation experiment on mouse embryonic stem cells (ESCs) revealed that only 3% of METTL3-knockout ESCs generated beating cardiomyocytes, whereas 50% of the control cells generated beating cardiomyocytes (Batista et al., 2014; Table 1). The m6A demethylase ALKBH5 is also involved in ESC-directed differentiation of cardiomyocytes. Thus, upregulating the level of RNA m6A methylation by inhibiting the expression of ALKBH5 can cause cardiomyocyte differentiation disorder. In contrast, overexpression of ALKBH5 inhibits this differentiation (Han et al., 2021c; Table 1). m6A RNA-binding proteins are also crucial in the differentiation of stem cells into cardiomyocytes. For example, knockout of YTHDF1 in mouse embryos severely impairs cardiomyocyte differentiation and downregulates cardiomyocyte-specific gene expression (Wang S. et al., 2021; Table 1). In contrast, knockout of YTHDF3 accelerates the differentiation of stem cells into cardiomyocytes by promoting the expression of cardiomyocyte-specific genes (Wang S. et al., 2021; Table 1). Thus, YTHDF1 and YTHDF3 play opposing roles in the differentiation of stem cells into cardiomyocytes, wherein YTHDF3 regulates cell differentiation by partially inhibiting the action of YTHDF1 (Wang S. et al., 2021). Therefore, m6A methylation regulates cell differentiation in the cardiovascular system, and its absence can lead to severe differentiation disorders.

### 3.3 Regulation of Cell Autophagy

Autophagy, in which m6A methylation modification plays a role, protects the heart from damage by inhibiting cardiomyocyte apoptosis (Saito et al., 2019). Ischemia-reperfusion (I/R) increases the level of m6A methylation in the mRNA of the transcription factor EB (TFEB) by inducing the expression of METTL3. This accelerates the degradation of the TFEB mRNA and inhibits TFEB expression, impairs autophagic flux, and promotes apoptosis (Song et al., 2019). In addition, m6A methylation regulates autophagy via miRNAs. In H/R-treated endothelial cells, METTL3 recruits DGCR8 to bind to pri-miR-20b in an m6A-dependent manner, promoting maturation of pri-miR-20b. The mature mic-20b consequently inhibits H/R-induced autophagy by inhibiting unc-51-like kinase 1 (ULK1) expression (Lu et al., 2020; Table 1). In contrast, FTO can directly reverses m6A methylation on ULK1, thereby prolonging the half-life of the ULK1 mRNA and promoting autophagy (Jin et al., 2018; Table 1). Notably, regulation of autophagy by m6A methylation is tissue- and disease-specific. For example, m6A methylation in CVDs inhibits H/R-induced autophagic flux, but in other diseases, m6A methylation promotes autophagy by accelerating its initiation (Chen X. et al., 2021). Indeed, the effect that m6A methylation has on autophagy depends on the target mRNA it modifies. Therefore, mapping the landscape of m6A-methylated target mRNAs can help to further explore the mechanism of m6A methylation in autophagy.

### 3.4 Regulation of Cell Apoptosis

Currently, studies investigating the role of m6A methylation in the regulation of apoptosis have presented contradictory observations. For instance, METTL3 enhances the binding of miR-25-3P and miR-873-5p to DGCR8 in an m6A-dependent manner to promote their maturation, which in turn activates the P13K/Akt pathway in cardiomyocytes and reduces H/R-induced apoptosis in cardiomyocytes (Zhao X. et al., 2021; Table 1). Interestingly, overexpression of FTO promotes the expression of Mhrt by downregulating m6A modification of Mhrt and inhibits the H/R-induced apoptosis of cardiomyocytes (Shen W. et al., 2021; Table 1). METTL3 and FTO have opposing effects on cardiomyocyte apoptosis, likely because of differential regulation of m6A methylation in different target mRNAs. Furthermore, studies have confirmed that targeting point mutations in the m6A modification sites of target transcripts using gene editing technologies can regulate apoptosis (Hao et al., 2020); thus, m6A methylation is a potentially effective means of regulating apoptosis. However, target transcripts that affect apoptosis first need to be identified.

### 3.5 Regulation of Inflammatory Cells

m6A methylation affects the autoimmunity and inflammatory response by regulating inflammatory cells, such as macrophages (Bechara and Gaffen, 2021). For instance, ALKBH5 inhibits the nuclear export of interleukin-6 (IL6) mRNA by demethylation, thus downregulating an inflammatory response (Zhao J. et al., 2020; Table 1). On the contrary, METTL3 promotes an inflammatory response induced by oxidized low-density lipoprotein (ox-LDL) by upregulating the m6A methylation of STAT1 (Li et al., 2022; Table 1). Similar to METTL3 overexpression, FTO silencing induces the transformation of macrophages into M1-type proinflammatory macrophages (Hu et al., 2019), thereby promoting an inflammatory response (Table 1). At present, the role and mechanisms of m6A methylation in regulating inflammation are still being studied. What we know is that MAPK and NF-κB inflammatory signaling pathways are targets of m6A methylation (Yu et al., 2019); With the progress of research, many other inflammatory signaling pathways regulated by m6A methylation will be found.

## 4 m6A Methylation and CVDs

CVDs, including cardiac and vascular diseases, are the leading cause of death i humans. As mentioned above, m6A affects the pathophysiology of CVDs by regulating cell proliferation, differentiation, autophagy, apoptosis, and inflammation (Song et al., 2019; Wang Y.-J. et al., 2021). Therefore, regulation of m6A methylation may possibly reverse the progression and provide new treatment strategies for CVDs. m6A sequencing data is crucial to screen key m6A target genes and we have summarize the publicly available m6A sequencing data in Gene Expression Omnibus (GEO) related to CVD in Table 2.

**TABLE 2:** m6A sequencing and microarray data in GEO related to CVD.

Study	Platform	Technology	Country	Year	Organism	Sample	CVD
GSE201764	GPL24247 Illumina NovaSeq 6,000 (*Mus musculus*)	high throughput sequencing	China	01 May 2022	*Mus musculus*	RNA m6A methylation between hypertrophic and normal mouse hearts	Heart failure
GSE189593	GPL25947 Illumina NovaSeq 6,000 (*Rattus norvegicus*)	high throughput sequencing	China	18 Feb 2022	*Rattus norvegicus*	m6A-modified transcripts between sham operation and MI rat models	Coronary heart disease
GSE159309	GPL25916 Arraystar Rat Epitranscriptomic microarray	m6A mRNA epitranscriptomic microarray	China	18 Aug 2021	*Rattus norvegicus*	m6A-modified transcripts between a control group and an lipopolysaccharide (LPS)-induced septic cardiomyopathy group	Septic cardiomyopathy
GSE159243	GPL11154 Illumina HiSeq 2000 (*Homo sapiens*)	high throughput sequencing	United States	01 Nov 2020	*Homo sapiens*	m6A-modified transcripts between human failing hearts and non-failing hearts	Heart failure
GSE131296	GPL11154 Illumina HiSeq 2000 (*Homo sapiens*)	high throughput sequencing	Germany	16 May 2019	*Mus musculus Homo sapiens*	RNA m6A methylation between hypertrophic and normal mouse hearts m6A-modified transcripts between human failing hearts and non-failing hearts	Heart failure
	GPL13112 Illumina HiSeq 2000 (*Mus musculus*)						
GSE112789	GPL17021 Illumina HiSeq 2,500 (*Mus musculus*)	high throughput sequencing	United States	13 Aug 2018	*Mus musculus* www.ncbi.nlm.nih.gov/Taxonomy/Browser/wwwtax.cgi?mode = Infoandid = 10,090"/>	RNA m6A methylation between healthy mouse hearts and failing mouse hearts	Heart failure
GSE147028	GPL24676 Illumina NovaSeq 6,000 (*Homo sapiens*)	high throughput sequencing	China	17 Mar 2020	*Homo sapiens*	RNA m6A methylation in aortic dissection and normal human aorta	Aortic dissection
GSE171371	GPL24247 Illumina NovaSeq 6,000 (*Mus musculus*)	high throughput sequencing	China	09 Apr 2021	*Mus musculus* www.ncbi.nlm.nih.gov/Taxonomy/Browser/wwwtax.cgi?mode = Infoandid = 10,090"/>	RNA m6A methylation in the aorta walls of AngII-induced abdominal aortic aneurysm model and normal mice	Aortic aneurysm

### 4.1 m6A Methylation and Vascular Diseases

Vascular diseases include atherosclerosis, pulmonary hypertension, and aortic aneurysm/dissection. As mentioned above, m6A methylation plays great role in the pathophysiology of vascular diseases, such as regulating endothelial cell proliferation, inflammation, and VSMC proliferation. While research on m6A methylation in vascular diseases is still in its infancy, m6A methylation seems to be a potential treatment target for vascular diseases.

#### 4.1.1 m6A Methylation and Atherosclerosis

Atherosclerosis, the primary culprit of cardiovascular disease, is characterized by the formation of atherosclerotic or fibrous plaques in the arterial intima of primarily large and medium-sized arteries. And for now, the mechanism of arteriosclerosis is still unclear. Recently, many studies have confirmed that the level of m6A methylation were significantly changed and it can delay the progression of atherosclerosis through the post-transcriptional regulation of RNA.(Zhang BY. et al., 2020; Guo et al., 2020; Jian et al., 2020; Chien et al., 2021; Gong et al., 2021).

**FIGURE 2:**
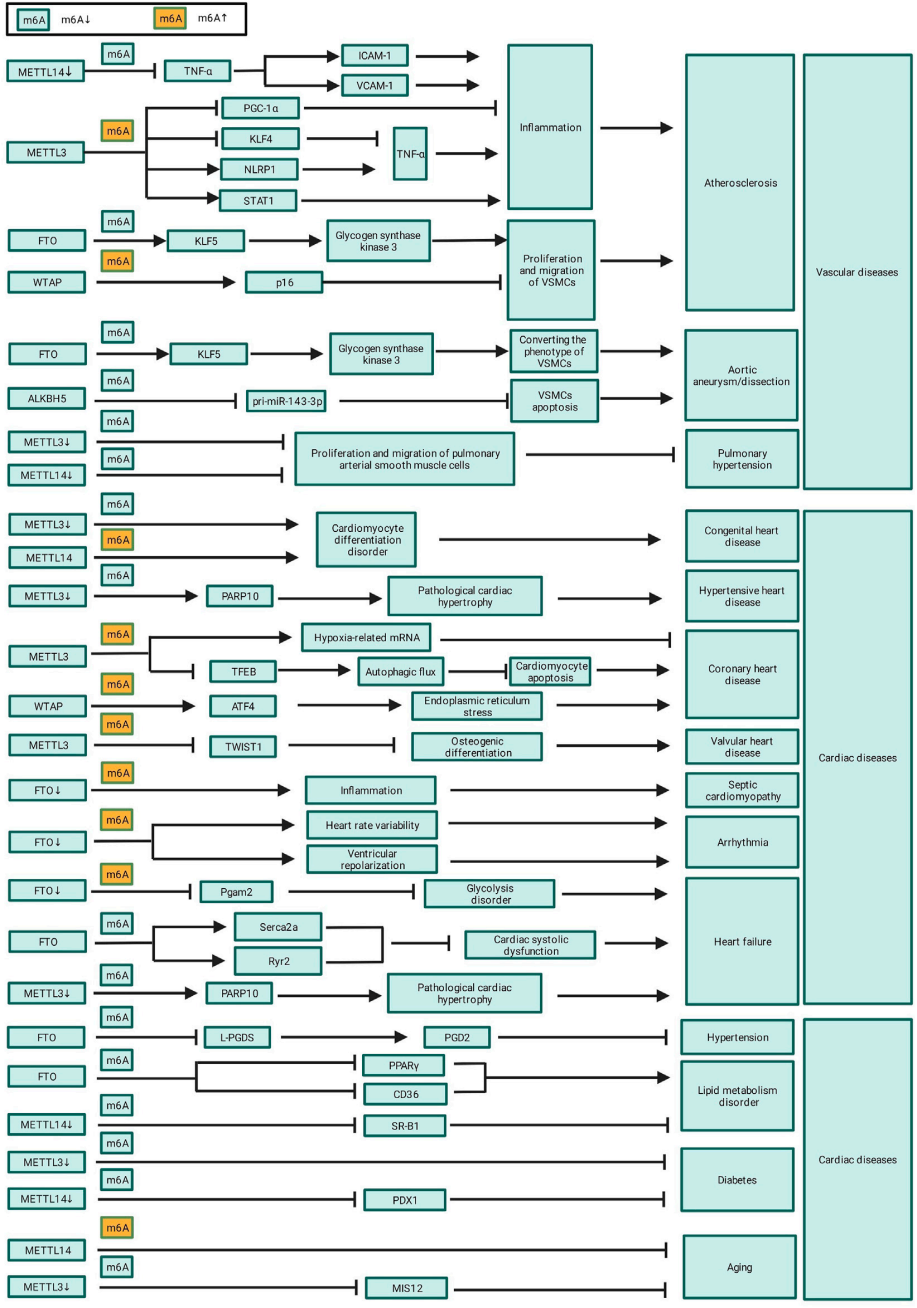
The role of m6A in CVD.

The inflammation of endothelial cell is an initial factor in the development of coronary atherosclerosis. Jian et al. found significantly increased RNA m6A modification levels in a tumor necrosis factor (TNF)-α-induced inflammation model of endothelial cell through up-regulation of METTL14. Moreover, they identified that the ablation of METTL14 significantly inhibited the expression of TNF-α-induced inflammatory factors, such as intercellular adhesion molecule-1 (ICAM-1) and vascular cellular adhesion molecule-1 (VCAM-1) (Jian et al., 2020; Table 3 and Figure 2). Zhang et al., meanwhile, found that METTL3 aggravated the inflammatory response of monocytes and promoted monocyte–endothelial cell adhesion by inducing the degradation of peroxisome proliferators-activated receptor γ coactivator l alpha (PGC-1α) mRNA in an m6A dependent way (Zhang X. et al., 2021; Table 3 and Figure 2). Chien et al. discovered that METTL3 up-regulated nucleotide-binding domain leucine-rich repeat pyrin domain containing 1 (NLRP1) and down-regulated Kruppel-like factor 4 (KLF4) through m6A modification of NLRP1 and KLF4 mRNA, and effectively promoted TNF-α-mediated inflammation in endothelial cells (Chien et al., 2021; Table 3 and Figure 2). Moreover, Li et al. and Liu et al. both reported that METTL3 modified signal transducer and activator of transcription 1 (STAT1) coding sequence and the 3’ UTR via m6A modification, thereby improving STAT1 mRNA stability, upregulating STAT1 protein levels, and promoting the M1 polarization of macrophages and inflammatory response (Liu Y. et al., 2019; Li et al., 2022; Table 3 and Figure 2).

**TABLE 3:** The role of m6A methylation in CVD.

CVD	m6A-Related molecules	Function	Target gene	Mechanism	References
Atherosclerosis	METTL14	Writer	—	ablation of METTL14 significantly inhibited the expression of TNF-α-induced inflammatory factors, such as ICAM-1 and VCAM-1	Jian et al. (2020)
	METTL3	Writer	PGC-1α	METTL3 aggravated the inflammatory response of monocytes and promoted monocyte–endothelial cell adhesion by inducing the degradation of PGC-1α mRNA in an m6A dependent way	Zhang et al. (2021d)
	METTL3	Writer	NLRP1	METTL3 up-regulated NLRP1 and down-regulated KLF4 through m6A modification of NLRP1 and KLF4 mRNA, and effectively promoted TNF-α-mediated inflammation in endothelial cells	Chien et al. (2021)
			KLF4		
	METTL3	Writer	STAT1	METTL3 promoted the M1 polarization of macrophages and inflammatory response by improving STAT1 mRNA stability	Liu et al. (2019b); Li et al. (2022)
	FTO	Eraser	KLF5	FTO decreases the m6A modification of KLF5 mRNA and promotes its expression, which further contributes to VSMC migration and promotes the phenotypic conversion of VSMC from a contractile to a proliferative phenotype	Wang et al. (2021b)
	WTAP	Writer	p16	WTAP upregulates the expression of p16 in an m6A-dependent manner, thereby inhibiting the proliferation and migration of VSMCs and delaying atherosclerotic progression	Wu et al. (2021)
Aortic aneurysm/dissection	FTO	Eraser	KLF5	FTO upregulates KLF5 expression through m6A methylation of KLF5 mRNA; KLF5 upregulates the expression of glycogen synthase kinase 3 and further promotes the development of aortic aneurysms by converting the contractile phenotype of VSMCs to a proliferative phenotype	Ma et al. (2020)
	ALKBH5	Eraser	pri-miR-143-3p	ALKBH5 can promote VSMCs apoptosis and facilitate the progression of aortic dissection via inhibition of the maturation of pri-miR-143-3p in an m6A-dependent manner	Wang et al. (2021b)
Pulmonary hypertension	METTL3 METTL14	Writer	—	knockdown of METTL3 and METTL14 can delay the progression of pulmonary hypertension by inhibiting the proliferation and migration of pulmonary arterial smooth muscle cells	Zhou et al. (2021b)
		Writer	—		
Congenital heart disease	METTL3	Writer	—	down-regulation of m6A level by ablation of METTL3 or overexpression of ALKBH5 inhibits the differentiation of mesodermal cells into cardiomyocytes	Zhao et al. (2021b)
	ALKBH5	Eraser	—		
Hypertensive heart disease	METTL3	Writer	PARP10	knockout of METTL3 upregulates PARP10 expression via down-regulation of m6A methylation of Parp10 mRNA, and accelerates pathological cardiac hypertrophy	Gao et al. (2020)
Coronary heart disease	METTL3		Hypoxia-related mRNA	METTL3 enhance the translation of hypoxia-related mRNA in hypoxic cardiomyocytes by up-regulating m6A methylation at the 5′-UTR of these mRNA, which can alleviate hypoxia-induced injury	Ye et al. (2021)
	METTL3		TFEB	METTL3 reduces the stability of TFEB mRNA by up-regulating m6A methylation of TFEB mRNA in hypoxic cardiomyocytes, which further inhibits the autophagic flux of hypoxic cardiomyocytes and promotes cardiomyocyte apoptosis, aggravating hypoxia-induced injury	Song et al. (2019)
	WTAP	Writer	ATF4	WTAP enhances the expression of ATF4 and promotes endoplasmic reticulum stress and ischemia/reperfusion injury by upregulating m6A methylation at the 5′-UTR of ATF4	Wang et al. (2021a)
Valvular heart disease	METTL3	Writer	TWIST1	METTL3 can promotes osteogenic differentiation of human aortic valve interstitial cells by inhibiting TWIST1 expression through an m6A-dependent pathway, aggravating valve calcification and leading to the development of valvular heart disease	Zhou et al. (2021a)
Septic cardiomyopathy	FTO	Eraser		FTO knockout aggravated inflammation and left ventricular dysfunction in an m6A-dependent manner in septic mice	Dubey et al. (2022)
Arrhythmia	FTO	Eraser		FTO knockout increased heart rate variability and altered ventricular repolarization in mice, leading to a potentially proarrhythmic remodeling of electrical and structural properties of the heart	Carnevali et al. (2014)
Heart failure	FTO		Pgam2	decreased FTO expression in failing heart increased m6A methylation of Pgam2 mRNA and promote its degradation, which further impair glycolysis process of myocardium tissue and cardiac systolic function	Zhang et al. (2021a)
	FTO		Serca2a	FTO also improve cardiac systolic function by increasing the expression of selective contractile transcripts, such as Serca2a or Ryr2, by up-regulation of m6A methylation of their mRNA	Mathiyalagan et al. (2019)
			Ryr2		
	METTL3		PARP10	METTL3 knockdown also accelerates heart failure progression by promoting pathological cardiac hypertrophy through upregulating the expression of PARP10	Dorn et al. (2019)
Hypertension	FTO		L-PGDS	FTO inhibits the expression of L-PGDS in an m6A-dependent manner and blocks synthesis of PGD2, which further increase vascular resistance and promote the development of hypertension	Krüger et al. (2020)
Lipid metabolism disorder	FTO		PPARγ	Overexpression of FTO reduced plasma total cholesterol levels and oxLDL deposition in macrophages, and increased cholesterol efflux from macrophages/foam cells by inhibiting the expression of PPARγ and CD36, alleviating lipid metabolism disorder	Mo et al. (2017)
			CD36		
	METTL14		SR-B1	down-regulation of METTL14 inhibit the expression of SR-B1 by downregulating the m6A methylation of SR-B1 mRNA, and further reduces cholesterol efflux and promotes foam cell formation, aggravating lipid metabolism disorder	Wu et al. (2021)
Diabetes	METTL3		Glucose metabolic genes	down-regulation of m6A level through METTL3 ablation can improve glucose tolerance and insulin sensitivity and delay the progress of diabetes by altering the expression of glucose metabolic genes	Li et al. (2020)
	METTL14		PDX1	down-regulation of m6A in pancreatic β-cells through METTL14 ablation impairs insulin secretion by decreasing PDX1 protein levels	De Jesus et al. (2019)
Aging	METTL3		MIS12	Down-regulation of m6A by knockout of METTL3 decreased the expression of MIS12 mRNA and accelerated cell ageing whereas up-regulation of m6A by overexpression of METTL3 delayed the process of cell ageing	Wu et al. (2020b)
	METTL14			Up-regulation of m6A by overexpression of METTL14 can attenuates cell aging	Zhang et al. (2020b)

m6A modification also participates in the progress of atherosclerosis through regulating the proliferation, migration, and phenotypic transformation of VSMCs ^12^. Specifically, FTO promotes the expression of *Krueppel-like factor 5* (*KLF5*) by decreasing the m6A modification of *KLF5* mRNA; this consequently upregulates the downstream expression of glycogen synthase kinase 3. This, in turn, contributes to VSMC migration and promotes the phenotypic conversion of VSMCs from a contractile to a proliferative phenotype, thereby promoting the development of atherosclerosis (Wang P. et al., 2021; Table 3 and Figure 2). Conversely, WTAP upregulates the expression of *p16* in an m6A-dependent manner, thereby inhibiting the proliferation and migration of VSMCs and delaying atherosclerotic progression (Wu et al., 2021; Table 3 and Figure 2).

#### 4.1.2 m6A Methylation and Aortic Aneurysm/Dissection

Compared to normal human, the level of m6A methylation in aortic tissues of aortic aneurysm patients is significantly upregulated. This high level of m6A methylation is associated with an increased risk of aortic aneurysm rupture, indicating that m6A methylation is involved in the development and prognosis of aortic aneurysm and dissection (He et al., 2019). m6A methylation is possibly involved in the progression of aortic dissection via regulation of smooth muscle cell (Li T. et al., 2021). Ma et al. found that FTO upregulates KLF5 expression through m6A methylation of KLF5 mRNA; KLF5 upregulates the expression of glycogen synthase kinase 3 and further promotes the development of aortic aneurysms by converting the contractile phenotype of VSMCs to a proliferative phenotype (Ma et al., 2020; Table 3 and Figure 2). Wang J. et al. (2021) found that ALKBH5 can promote VSMCs apoptosis and facilitate the progression of aortic dissection via inhibition of the maturation of pri-miR-143-3p in an m6A-dependent manner (Wang P. et al., 2021; Table 3 and Figure 2).

#### 4.1.3 m6A Methylation and Pulmonary Hypertension

Several studies have reported that m6A methylation levels are significantly upregulated in a rat model of pulmonary hypertension, and these differentially m6A methylated genes proved to be involved in inflammation, glycolysis, endothelial cell receptor activation, and lung development, which can accelerate the progress of pulmonary hypertension (Xu et al., 2021; Zeng et al., 2021). Down-regulation of m6A modification by knockdown of METTL3 and METTL14 can delay the progression of pulmonary hypertension by inhibiting the proliferation and migration of pulmonary arterial smooth muscle cells (Zhou X.-L. et al., 2021; Table 3 and Figure 2). In addition, m6A methylation was also found to affect the progression of pulmonary hypertension through the circRNA-miRNA-mRNA network, and two pulmonary hypertension-related circRNAs, circXpo6 and circTmtc3, were identified (Su et al., 2020). Of note, m6A methylation is a biomarker of epigenetic modifications in key genes that regulate pulmonary arterial pressure and lung development; however, further study is needed to characterize the specific regulatory mechanisms underlying the involvement of m6A methylation in pulmonary hypertension.

### 4.2 m6A Methylation and Cardiac Diseases

Cardiac diseases include congenital, hypertensive, atherosclerotic, and valvular heart diseases as well as cardiomyopathy, arrhythmia, and heart failure. As mentioned above, m6A methylation play a crucial role in the pathogenesis of cardiac diseases. Currently, the landscape of m6A modifications in hypertensive heart disease and heart failure patients has been mapped; thus, future research needs to focus on mapping the landscape and modification patterns in other cardiac diseases, in order to identify key transcripts that regulate the progression of cardiac diseases.

#### 4.2.1 m6A Methylation and Congenital Heart Disease

The heart is developed from mesodermal cells during embryonic development; abnormal differentiation of embryonic stem cells lead to congenital heart disease (Kattman et al., 2007; Bu et al., 2009). m6A methylation has been proved to be a critical role in embryonic stem cell differentiation and cardiomyocyte proliferation. Additionally, m6A methylation is a key regulator of maintaining the pluripotency of embryonic stem cells, reprogramming of somatic cells, and differentiation and proliferation of stem and progenitor cells (Malla et al., 2019). As previously mentioned, down-regulation of m6A level by ablation of METTL3 or overexpression of ALKBH5 inhibits the differentiation of mesodermal cells into cardiomyocytes (Table 3; Figure 2). Therefore, there is a link between m6A methylation and congenital heart diseases. Exploring the specific role and therapeutic potential of m6A methylation in the progression of congenital heart disease will help to the prevention and treatment of congenital heart disease.

#### 4.2.2 m6A and Hypertensive Heart Disease

Hypertensive heart disease is charactered by left ventricular hypertrophy and heart failure caused by uncontrolled hypertension. Cardiomyocyte hypertrophy is an adaptive response to afterloads caused by uncontrolled hypertension; however, persistent cardiomyocyte hypertrophy induces cardiomyocyte apoptosis, necrosis, and fibrotic proliferation, ultimately causing heart failure (Krüger et al., 2020).

Some studies have shown that the percentage of m6A-methylated RNA was significantly increased in hypertension-induced hypertrophic cardiomyocytes compared to that of healthy cardiomyocytes (Dorn et al., 2019; Berulava et al., 2020) and that the degree of cardiac hypertrophy is positively correlated with the level of m6A methylation (Dorn et al., 2019; Chen Y.-S. et al., 2021). Overexpression of METTL3 can induce compensatory cardiomyocyte hypertrophy without deteriorating cardiac function (Dorn et al., 2019), whereas knockout of METTL3 upregulates PARP10 expression via down-regulation of m^6^A methylation of *Parp10* mRNA, and accelerates pathological cardiac hypertrophy (Gao et al., 2020; Table 3 and Figure 2). Down-regulation of m6A might be one of the mechanisms by which hypertension-induced pathological cardiac hypertrophy and heart failure.

#### 4.2.3 m6A Methylation and CHD

CHD, also called ischemia heart disease, is characterized by a reduction of oxygen supply due to coronary atherosclerosis. Shen et al. found that their is substantial difference between the transcriptomes and proteomes of hypoxic cardiomyocytes, implying epigenetic modifications of RNA occurred in hypoxic cardiomyocytes (Shen et al., 2020).

Rapid gene expression after hypoxia stress is required to prevent cardiomyocytes from the hypoxia-induced injury. As the most abundant RNA modification, m6A methylation is dramatically up-regulated in cardiomyocytes after hypoxia exposure (Fry et al., 2017; Chokkalla et al., 2019; Ye et al., 2021). Ye et al. reported that m6A methylation at the 5′-UTR of hypoxia-related mRNA is upregulated via overexpression of METTL3 induced by hypoxia; and this promote the localization of eIF4A2 to target genes and enhance the translation of these genes in hypoxic cardiomyocytes, which can alleviate hypoxia-induced injury (Ye et al., 2021; Table 3 and Figure 2). However, up-regulation of m6A methylation maybe a double-edged sword after hypoxia exposure. Song et al. found that the METTL3 reduces the stability of TFEB mRNA by up-regulating m6A methylation of TFEB mRNA in hypoxic cardiomyocytes, which further inhibits the autophagic flux of hypoxic cardiomyocytes and promotes cardiomyocyte apoptosis, aggravating hypoxia-induced injury (Song et al., 2019). Activation of transcription factor 4 (ATF4) enhances the expression of endoplasmic reticulum stress-related genes and contributes to myocardial ischemia/reperfusion injury by promoting endoplasmic reticulum stress. The m6A methylation at the 5′-UTR of ATF4 is up-regulated through increased WTAP expression induced by ischemia/reperfusion injury and this promote the binding of eIF3A to ATF4, which further enhances the expression of ATF4 and promotes endoplasmic reticulum stress and ischemia/reperfusion injury (Wang J. et al., 2021).

#### 4.2.4 m6A Methylation and Valvular Heart Disease

Redifferentiation of interstitial cells in the human aortic valve into osteoblast-like cells is an important mechanism of aortic valve calcification, which is a primary cause of heart valve disease. METTL3 can promotes osteogenic differentiation of human aortic valve interstitial cells by inhibiting twist-related protein 1 (TWIST1) expression through an m6A-dependent pathway, aggravating valve calcification and leading to the development of valvular heart disease (Zhou T. et al., 2021). At present, there are only a few studies on the role of m6A methylation in valvular heart disease. Thus, further research is needed to decipher the exact mechanism and therapeutic value of m6A methylation in this disease.

#### 4.2.5 m6A Methylation and Septic Cardiomyopathy

Approximately, septic cardiomyopathy occurs in 50% of septic shock patients. Moreover, cardiac dysfunction caused by septic cardiomyopathy is a key cause of high mortality in these patients (Zaky et al., 2014). m6A methylation pattern in cardiac tissue of septic shock rat model was found to be significantly different from control rat. Additionally, Gene Ontology (GO) and Kyoto Encyclopedia of Genes and Genomes (KEGG) analyses showed that differentially m6A-methylated mRNAs are mainly involved in interleukin-17 (IL17) signaling pathways, TNF signaling pathway, and cytokine receptor interaction pathway, suggesting that m6A methylation possibly participate in the progression of septic cardiomyopathy through immune and inflammatory response (Han Y.-C. et al., 2021; Shen Z.-J. et al., 2021). FTO was proved to be a significant regulator of the m6A methylation level in the myocardial tissue of septic mice. FTO knockout aggravated inflammation and left ventricular dysfunction in an m6A-dependent manner in septic mice (Dubey et al., 2022). Therefore, m6A methylation may be a potential target for alleviating cardiac dysfunction in septic shock patients.

#### 4.2.6 m6A Methylation and Arrhythmia

To date, only a few studies explored the relationship between m6A modifications and arrhythmias. In one study, m6A demethylase FTO knockout increased heart rate variability and altered ventricular repolarization in mice, leading to a potentially proarrhythmic remodeling of electrical and structural properties of the heart (Table 3; Figure 2). It is suggested that m6A methylation can make the mice more prone to stress-induced tachyarrhythmias (Carnevali et al., 2014). However, further research is required to identify the m6A-regulated target genes associated with arrhythmia and to assess the involvement of other m6A methyltransferases and demethylases.

#### 4.2.7 m6A Methylation and Heart Failure

Extensive studies have confirmed that the level of m6A methylation is significantly upregulated. METTL3 and FTO were proved to be the key regulators of m6A methylation in failure heart. The expression of METTL3 is increased while that of FTO is reduced; the synergistic function of METTL3 and FTO up-regulate the level of m6A methylation in failing hearts (Hinger et al., 2021). GO and KEGG pathway analysis show that these differential m6A methylation modifications are not randomly distributed but actually enriched in certain mRNA subsets, including mRNAs involved in glycolytic metabolism, mitochondrial function, myocardial fibrosis and cardiac contractility (Berulava et al., 2020; Zhang et al., 2021a; Hinger et al., 2021).

Glycolysis is essential to myocardial energy metabolism; myocardial energy metabolic disorders would directly affect cardiac systolic function. In a transverse aortic constriction (TAC)-induced heart failure model, Zhang et al. found that decreased FTO expression in failing heart increased m6A methylation of *Pgam2* mRNA and promote its degradation, which further impair glycolysis process of myocardium tissue and cardiac systolic function (Zhang et al., 2021a). FTO also improve cardiac systolic function by increasing the expression of selective contractile transcripts, such as Serca2a or Ryr2, by up-regulation of m6A methylation of their mRNA (Mathiyalagan et al., 2019). These suggest FTO is a potential therapeutic in the treatment of heart failure.

However, METTL3 knockdown also accelerates heart failure progression by promoting pathological cardiac hypertrophy through upregulating the expression of PARP10 as mentioned above (Dorn et al., 2019). This may be explained by the fact that METTL3 and FTO participate in the progression of heart failure by regulating m6A methylation of target transcriptions rather regulating than the global level of m6A methylation. The target transcriptions of METTL3 or FTO determine their roles of in the progression of heart failure.

### 4.3 M6A Methylation and CVD Risk Factors

#### 4.3.1 m6A Methylation and Hypertension

A preliminary understanding on how m6A methylation affects the development of hypertension is primarily based on the high-throughput sequencing data of hypertension patients. Mo et al. identified more m6A-single nucleotide polymorphism (m6A-SNP) loci in hypertension patients than in healthy individuals. Remarkably, they identified 1,236 hypertension-specific m6A-SNPs (Mo et al., 2019). Wu et al. found that coding regions of mRNA were enriched with m6A modifications in hypertensive rats. Additionally, compared to those of control mice, hypertensive mice exhibited significant differences in the number of m6A modifications. Furthermore, GO and KEGG pathway analyses revealed that m6A-modified genes are involved in inflammation, proximal tubule development, RNA methyltransferase activity, water channel activity, actin cytoskeleton pathway regulation, and neuroligand receptor activity.

Krüger et al. (2020) revealed that FTO-mediated m6A demethylation plays a key role in regulating arterial myogenic contraction and vascular resistance. Prostaglandin D2 (PGD2) activates G protein-coupled receptors expressed on vascular smooth muscle membranes to promote the relaxation of VSMCs, delaying the progression of hypertension. Lipocalin-type prostaglandin D synthase (L-PGDS) is the main enzyme to synthesize PGD2. In human and mouse blood vessels, FTO inhibits the expression of L-PGDS in an m6A-dependent manner and blocks synthesis of PGD2, which further increase vascular resistance and promote the development of hypertension (Krüger et al., 2020).

#### 4.3.2 m6A Methylation and Lipid Metabolism Disorder

Disorders of lipid metabolism are common drivers of atherosclerosis. Notably, m6A methylation affects blood lipid levels and elevates blood lipid deposition, aggravating atherosclerotic progression (Davis et al., 2014; Chedraui et al., 2016). Overexpression of FTO reduced plasma total cholesterol levels and ox-LDL deposition in macrophages, and increased cholesterol efflux from macrophages/foam cells by inhibiting the expression of peroxisome proliferator-activated receptor γ (PPARγ) and cluster of differentiation 36 (CD36), alleviating lipid metabolism disorder (Mo et al., 2017; Table 3 and Figure 2). Wu et al. found that down-regulation of METTL14 inhibit the expression of scavenger receptor B-type 1 (SR-B1) by downregulating the m6A methylation of SR-B1 mRNA, and further reduces cholesterol efflux and promotes foam cell formation, aggravating lipid metabolism disorder (Wu et al., 2021; Table 3 and Figure 2).

#### 4.3.3 m6A Methylation and Diabetes

Li et al. found that m6A level is increased in diabetes mice model due to upregulation of METTL3 induced by diabetes. To verify the role of m6A in diabetes, they constructed METTL3 knockout mice and confirmed that down-regulation of m6A level through METTL3 ablation can improve glucose tolerance and insulin sensitivity and delay the progress of diabetes by altering the expression of glucose metabolic genes (Li et al., 2020). However, different from the findings of Li et al., Jesus et al. verified that m6A level and METTL14 expression in pancreatic β-cells of diabetes patients is decreased, and down-regulation of m6A in pancreatic β-cells through METTL14 ablation impairs insulin secretion by decreasing pancreatic and duodenal homeobox 1 (PDX1) protein levels (De Jesus et al., 2019). This difference can be interpreted with the poor conservatism of m6A modification between different organs. But they both confirmed that m6A modification is involved in the progress of diabetes. Further studies are needed to explore the mechanism of m6A modification involved in the progress of diabetes.

#### 4.3.4 m6A Methylation and Aging

Aging is an important risk factor of CVD. Numerous reports have explored the relationship of m6A modification and aging, and found that m6A modification participated in the ageing process. Wu et al. found that the level of m6A and METTL3 is reduced in aged cells. Down-regulation of m6A by knockout of METTL3 decreased the expression of MIS12 mRNA and accelerated cell ageing whereas up-regulation of m6A by overexpression of METTL3 delayed the process of cell ageing (Wu Z. et al., 2020). Up-regulation of m6A by overexpression of METTL14 can also attenuates cell aging (Zhang J. et al., 2020). These studies show that m6A modification has the potential as a therapeutic target in the treatment of aging-related disease.

## 5 Prospects and Summary

Over the past 10 years, researchers have mapped the m6A methylation landscape in CVDs, such as heart failure and coronary heart disease, confirmed that regulation of m6A methylation can reverse pathophysiological processes. Additionally, there is a complicated interrelations between m6A and other epigenetics regulation layers like histone modifications and non-coding RNAs. For example, m6A demethylase ALKBH5 as mentioned above facilitate the progression of aortic dissection via inhibition of the maturation of pri-miR-143-3p in an m6A-dependent manner. The intricate crosstalk between m6A and other epigenetics regulation layers triggers epigenetic remodeling, further affecting the progression of CVDs. So, it is a promising direction to explore the relationship of m6A and other epigenetics regulation layers.

Another challenge is that m6A methylases and demethylases regulate m6A methylation of RNA without high-specificity. They also regulate the m6A methylation levels of other genes when they were used to regulate the metabolism of target transcriptions, which bring obstacles in the application of these m6A methylation regulators. However, studies using gene editing technologies to regulate the m6A modifications of a single target transcript have achieved remarkable results (Liu X.-M. et al., 2019; Rau et al., 2019), indicating that gene editing may be a more precise tool to regulate m6A methylation.

In all, we discussed the biological role of m6A methylation and its potential therapeutic role in CVDs. At present, research investigating the role of m6A methylation in the occurrence and development of CVDs is still in its infancy; however, future research may identify additional functions and underlying mechanisms of m6A methylation in CVDs, which will be a major advance in the field of epigenetics.

## Glossary

CVD: cardiovascular disease
m6A: n6-methyladenosine
mRNA: messenger RNA
tRNA: transfer RNA
circRNA: circular RNA: circular RNA
lncRNA: long non-coding RNA: long noncoding RNA
rRNA: ribosomal RNA
UTRs: untranslated regions
METTL 3: methyltransferase-like 3
METTL14: methyltransferase-like 14
WTAP: Wilms tumor 1-associated protein
SAM: S-adenosylmethionine
VIRMA: Vir-like m6A methyltransferase-associated protein
RBM15: RNA binding motif protein 15
HAKAI: Cbl photo oncogene like 1
ZC3H13: zinc finger CCCH-type containing 13
FTO: fat mass and obesity-associated protein
ALKBH5: ALKB homologue 5 protein
CHD: coronary heart disease
SRSF3: YTHDC, TH domain-containing protein; serine- and arginine-rich splicing factor 3
NXF1: nuclear RNA-export factor 1
IGF2BPs: insulin-like growth factor 2 mRNA-binding proteins
HNRNPC: heterogeneous nuclear ribonucleoprotein C
HNRNPA2B1: heterogeneous nuclear ribonucleoprotein A2/B1
MEPCE: methylphosphate capping enzyme
TREX: transcription–export
eIF: eukaryotic initiation factor
CCR4: carbon catabolite-repression 4
NOT: negative on TATA-less
HRSP12: heat-responsive protein 12
RNase: ribonuclease
MRP: mitochondrial RNA-processing
DGCR8: DiGeorge syndrome critical region 8
DROSHA: Drosha ribonuclease III
miRNA: microRNA
pri-miRNA: primary-miRNA
lncRNA: long non-coding RNA: long noncoding RNA
circRNA: circular RNA: circular RNA
VSMC: vascular smooth muscle cell
ESCs: embryonic stem cells
I/R: ischemia-reperfusion
TFEB: transcription factor EB
ULK1: unc-51-like kinase 1
IL6: interleukin-6
ox-LDL: oxidized-low density lipoprotein
GEO: Gene Expression Omnibus
TNF: tumor necrosis factor
ICAM-1: intercellular adhesion molecule-1
VCAM-1: vascular cell adhesion molecule-1
PGC-1α: peroxisome proliferators-activated receptor γ coactivator l alpha
NLRP1: nucleotide-binding domain leucine-rich repeat pyrin domain containing 1
KLF4: kruppel-like factor 4
STAT1: signal transducer and activator of transcription 1
KLF5: Krueppel-like factor 5
ATF4: activation of transcription factor 4
GO: Gene Ontology
KEGG: Kyoto Encyclopedia of Genes and Genomes
IL17: interleukin-17
TAC: transverse aortic constriction
m6A-SNP: m6A-single nucleotide polymorphism
PGD2: prostaglandin D2
L-PGDS: lipocalin-type prostaglandin D synthase
PPAR γ: peroxisome proliferator-activated receptor γ
CD36: cluster of differentiation 36
SR -B1: scavenger receptor B-type 1
PDX1: pancreatic and duodenal homeobox 1.
